# Glycopolymer-Wrapped Carbon Nanotubes Show Distinct Interaction of Carbohydrates With Lectins

**DOI:** 10.3389/fchem.2022.852988

**Published:** 2022-03-03

**Authors:** Ana M. DiLillo, Ka Keung Chan, Xue-Long Sun, Geyou Ao

**Affiliations:** ^1^ Department of Chemical and Biomedical Engineering, Washkewicz College of Engineering, Cleveland State University, Cleveland, OH, United States; ^2^ Department of Chemistry, Center for Gene Regulation in Health and Disease (GRHD), Cleveland State University, Cleveland, OH, United States

**Keywords:** carbon nanotubes, glycopolymers, wrapping conformation, protein interactions, optical sensing

## Abstract

Glyconanomaterials with unique nanoscale property and carbohydrate functionality show vast potential in biological and biomedical applications. We investigated the interactions of noncovalent complexes of single-wall carbon nanotubes that are wrapped by disaccharide lactose-containing glycopolymers with the specific carbohydrate-binding proteins. The terminal galactose (Gal) of glycopolymers binds to the specific lectin as expected. Interestingly, an increased aggregation of nanotubes was also observed when interacting with a glucose (Glc) specific lectin, likely due to the removal of Glc groups from the surface of nanotubes resulting from the potential binding of the lectin to the Glc in the glycopolymers. This result indicates that the wrapping conformation of glycopolymers on the surface of nanotubes potentially allows improved accessibility of the Glc for specific lectins. Furthermore, it shows that the interaction between Glc groups in the glycopolymers and nanotubes play a key role in stabilizing the nanocomplexes. Overall, our results demonstrate that nanostructures can enable conformation-dependent interactions of glycopolymers and proteins and can potentially lead to the creation of versatile optical sensors for detecting carbohydrate-protein interactions with enhanced specificity and sensitivity.

## Introduction

Elaborate molecular recognitions assisted by hydrophobic and electrostatic interactions and hydrogen bonding are ubiquitous in living systems (Reed et al., 1988; Miura et al., 2016). The recognition interactions between cell surface carbohydrates and carbohydrate-binding proteins (CBPs), such as lectins, play a vital role in a multitude of cellular activities, such as cellular adhesion, immune responses, and infections (Zhang et al., 2010; Zeng et al., 2012; Miura et al., 2016; Liyanage and Yan 2020). For example, the attachment of CBPs, including FimH and FaeG proteins of bacterial fimbriae, to glycoconjugates (i.e., glycolipids, glycoproteins, and proteoglycans) on the host cell surfaces initiates infections (Kato and Ishiwa 2015; Moonens et al., 2015; Baker and Jafri 2016). Due to the relatively weak interactions between monomeric saccharide and CBPs featuring hydrogen bonding and chelation with ions, biological systems employ a saccharide-assembly structure of oligosaccharides to amplify carbohydrate-protein interactions (Zhang et al., 2010; Cecioni et al., 2015; Bernardi et al., 2013). Therefore, developing biomimetic synthetic materials that feature multivalent carbohydrate-protein interactions has vast potential in biomedical applications, including biosensing, drug delivery, therapeutics, and pathogen inhibition (Zhang et al., 2010; Richards et al., 2012; Miura et al., 2016; Bristol et al., 2020).

Nanomaterials, including metal nanoparticles and nanocarbons (e.g., nanotubes and graphene), have been widely utilized as multivalent scaffolds for the significant amplification of carbohydrate-protein interactions due to their high specific surface area, distinct shape, and unique optical and physiochemical properties (Wang et al., 2010a; Chen et al., 2013; Qi et al., 2015; Liyanage and Yan 2020). Both covalent and noncovalent approaches are employed for functionalizing the surface of nanomaterials with high-density carbohydrate ligands, and the resulting complexes are known as glyconanomaterials. The noncovalent approach preserves the intrinsic properties of both nanomaterial scaffolds and carbohydrate ligands, while the covalent approach generally produces glyconanomaterials with high stabilities. Yan and colleagues have successfully demonstrated the coupling of nanomaterials (e.g., gold, iron oxide, and silica nanoparticles and graphene) with diverse structures of carbohydrate ligands primarily through covalent chemistry (Liu et al., 2009; Jayawardena et al., 2013; Wang et al., 2013; Kong et al., 2019). The resulting glyconanomaterials have shown significant increase in the binding affinity to specific lectins through forming crosslinked agglomerates, by up to several orders of magnitudes compared to the free carbohydrate ligands due to multivalent interactions (Liyanage and Yan 2020). The carbohydrate-protein interactions assisted by glyconanomaterials also revealed the important role of the spatial arrangement of ligands on the surface of nanomaterials in lectin binding (Wang et al., 2010b; Liyanage and Yan 2020). Additionally, the noncovalent complexation of nanocarbons through helical wrapping of nanotubes by polysaccharides bearing carbohydrate pendent groups and the adsorption of carbohydrate-containing molecules onto the surface of nanocarbons through hydrophobic interactions and *p*-π stacking have been reported previously (Hasegawa et al., 2004; Chen et al., 2006; Chen et al., 2012). However, studies on the specific interaction of glyconanomaterials and lectins so far have been generally focused on the currently known binding behavior of lectins to the terminal glycan of oligosaccharides (Ting et al., 2010; Rillahan and Paulson 2011).

We have previously reported the creation of stable and water-soluble glycopolymer-wrapped single-wall carbon nanotubes (Glyco-SWCNTs) through the noncovalent complexation of nanotubes with polymers, where the interaction of glycopolymers with SWCNTs is strongly dependent on the carbohydrate identity, ligand density, and the polymer chain length (Cantwell et al., 2020). Synthetic glycopolymers with highly tunable carbohydrate structures are chemically stable and can be used as glycoconjugate mimetics for many biological applications (Sun 2018; Chan et al., 2020). The multivalency of glycopolymers together with the tunability in polymer chain length, ligand composition and density, flexibility, and conformation promotes strong carbohydrate-mediated interactions with lectins (Kumar et al., 2011; Narla et al., 2012; Miura et al., 2016; Tang et al., 2017). Additionally, the wrapping of SWCNTs by less-flexible disaccharide lactose-containing glycopolymers produces stable complexes in aqueous environment while preserving the intrinsic optical, electronic, and physiochemical properties of nanotubes (Cantwell et al., 2020). Particularly, Glyco-SWCNT complexes fluoresce in the near-infrared (NIR) spectral regime, where attenuated tissue autofluorescence and deep tissue penetration occur in biological samples (Weisman and Bachilo 2003; Lin and Weisman 2016). The intrinsic NIR fluorescence of SWCNTs is an important feature of nanotube applications in biomedical applications, including biosensing, imaging, and therapeutics (Budhathoki-Uprety et al., 2019; Hu et al., 2020; Gravely and Roxbury 2021).

In this work, we further demonstrated the carbohydrate functionality of stable Glyco-SWCNT complexes that are wrapped by disaccharide lactose-containing homopolymers through interacting with specific lectins including lectins from *Arachis hypogaea* (peanut, PNA), *Canavalia ensiformis* (Concanavalin A, Con A), and *Galanthus nivalis* (snowdrop, GNA). We monitored the specific interactions between Glyco-SWCNTs and lectins through measuring the absorption and NIR fluorescence of SWCNTs as well as the visible fluorescence of fluorescein isothiocyanate (FITC) labels of lectins. Additionally, kinetics of carbohydrate-lectin interactions through monitoring the fluorescence spectral change of lectin FITC marker provided a fast and efficient way to probe the binding affinity of a specific lectin to Glyco-SWCNTs. More interestingly, the observed binding of Con A to Glyco-SWCNT complexes reveals the important role of the wrapping conformation of polymers around the surface of SWCNTs in enabling unique glyconanomaterial-lectin interactions.

## Materials and Methods

### SWCNT Sample Preparation

CoMoCAT SWCNT powder (SG65i-L39, CHASM Advanced Materials), that is enriched in small diameter (6,5) chirality species, was dispersed in a total volume of 1 ml aqueous solutions of synthetic glycopolymers by probe tip sonication (model VCX 130, Sonics and Materials, Inc.) in an ice bath at a power level of 8 W. Disaccharide *ß*-lactose (Lact)-containing homopolymers were synthesized from lactosylacrylamide (i.e., Lact-AM) monomer using cyanoxyl free radical-mediated polymerization (CFRMP) scheme in one-pot fashion as previously reported (Tang et al., 2017; Chan et al., 2020). The polymer chain lengths of Lact-homopolymers utilized here are 400 and 415 (i.e., Lact-AM 400 and Lact-AM 415), respectively. Based on the availability of synthetic polymers, we utilized Lact-AM 415 for the optimization of Glyco-SWCNT dispersions only and Lact-AM 400 for producing Glyco-SWCNTs for protein interaction experiments. Initially, 0.1 mg/ml SWCNTs were utilized to test the dispersion conditions including the sonication time and the mass ratios of SWCNTs:Lact-AM 415. For the sonication time test, samples with a fixed mass ratio of SWCNTs:Lact-AM 415 = 1:4 in deionized (DI) water were sonicated for 40 and 120 min on a continuous base and for 30 + 30, 45 + 45, and 40 + 40 + 40 min, respectively, where the plus sign indicates a 30-min rest between each sonication step. The sonication time of 45 + 45 min was found to be optimal and was used for testing varying mass ratios of SWCNTs:Lact-AM 415 = 1: n , where n = 0.5, 1, 2, 3, 4, and 8, respectively. After tip sonication, supernatant dispersions were collected after centrifugation at 17,000 g for 90 min at 19°C.

After determining the optimum dispersion condition, stock Glyco-SWCNT samples were prepared from 1 mg/ml SWCNTs at a mass ratio of SWCNTs:Lact-AM 400 = 1:4 following the sonication and supernatant collection steps described above for protein interaction experiments. Additionally, the control sample of DNA-wrapped SWCNTs (DNA-SWCNTs) was prepared by probe tip sonication using our previously reported method (Ao et al., 2016). Briefly, 1 mg/ml SWCNTs were mixed with a total volume of 1 ml aqueous solution of 2 mg/ml single-stranded DNA sequence ATTTATTATTTA (Integrated DNA Technologies) containing 0.1 mol/L NaCl, and tip sonicated in an ice bath for 2 h at power level of 8 W. Supernatant dispersions were collected after centrifugation at 17,000 g for 90 min at 19°C.

### Interaction of Proteins with Glycopolymer-SWCNT Complexes

Fluorescein isothiocyanate (FITC) conjugated PNA (lectin from *Arachis hypogaea* (peanut), ≈120 kDa) (PNA-FITC), FITC conjugated Concanavalin A (lectin from *Canavalia ensiformis* (Jack bean), type IV, ≈ 102 kDa) (Con A-FITC), GNA (lectin from *Galanthus nivalis* (snowdrop), ≈52 kDa), and bovine serum albumin (BSA, ≈ 66 kDa) were purchased from Sigma-Aldrich. Stock samples of Glyco-SWCNTs were diluted 8× times in pH 7.40 phosphate buffered saline (PBS) buffer solution for protein interaction experiments. Stock solutions of proteins were prepared by dissolving 1 mg/ml proteins in pH 7.40 PBS buffer solution. Small aliquots (i.e., 0.25 ± 0.03 to 38.64 ± 1.00 µL) of protein solutions were added to 100 µL Glyco-SWCNT samples in PBS buffer solution. The difference in the minor dilution of samples after adding various concentrations of proteins was accounted for when determining the SWCNT mass percentage remaining in the supernatants. All tests were examined by producing three repeats of each sample.

Glyco-SWCNT samples (using Lact-AM 400) at a nanotube concentration of 9.66 ± 0.74 μg/ml in PBS buffer solution were incubated with 38.64 ± 1.00 μg/ml (i.e., 0.32 ± 0.01 μM) of PNA-FITC at varying time periods of 0, 5, and 10 min, respectively, at room temperature to determine the incubation time needed (i.e., 5 min) for protein interactions. Then, Glyco-SWCNT samples of 100 µL volume at a nanotube concentration of 4.21 ± 0.13 μg/ml in PBS buffer solution were incubated with different proteins (i.e., PNA, Con A, GNA, and BSA) at varying concentrations from 0 to 1.50 ± 0.20 µM for 5 min at room temperature. The mixtures were then vortex mixed and centrifuged for 4 min at 17,000 g and 19°C to collect the supernatant samples for characterization.

Competitive and sequential binding tests of proteins with a volume of 100 µL Glyco-SWCNTs (using Lact-AM 400) at a nanotube concentration of 4.21 ± 0.13 μg/ml in PBS buffer solution were carried out at room temperature as following. The stock solutions were prepared at 1 mg/ml of lectins (i.e., PNA and Con A, respectively) and 10 mg/ml of free sugar *ß*-lactose in pH 7.40 PBS buffer solution, respectively. Small aliquots of lectins (i.e., 10.6 µL of PNA and 8.9 µL of Con A, respectively) and free sugar *ß*-lactose (i.e, 4.1 µL) were added to the Glyco-SWCNT samples. Competitive binding tests were performed by adding a mixture of 0.77 ± 0.01 µM lectin (i.e., PNA and Con A, respectively) and 0.36 ± 0.05 mg/ml of free sugar *ß*-lactose in PBS buffer solution simultaneously into Glyco-SWCNT samples. The amount of free sugar *ß*-lactose roughly corresponds to that of Lact group in the Lact-AM 400 homopolymer in a typical Glyco-SWCNT sample used for protein interactions. The mixture was incubated for 5 min followed by centrifugation for 4 min at 17,000 g and 19°C to collect supernatant for characterization. Sequential binding I was performed by first incubating Glyco-SWCNTs with 0.77 ± 0.01 µM lectin (i.e., PNA and Con A, respectively) for 5 min. This is followed by adding 0.36 ± 0.05 mg/ml of free sugar *ß*-lactose to the mixture by vortex mixing and incubating for additional 5 min before centrifugation and collection of supernatants as described previously. Lastly, sequential binding II was performed by mixing 0.36 ± 0.05 mg/ml of free sugar *ß*-lactose and 0.77 ± 0.01 µM lectin (i.e., PNA and Con A, respectively) and incubating for 5 min. The mixture was then added to Glyco-SWCNTs and allowed to incubate for additional 5 min before proceeding with centrifugation and collection of supernatants as described previously.

### Optical Spectroscopy Characterization

Optical spectroscopy characterization including vis-NIR absorbance and visible and NIR fluorescence measurements was performed on a NS3 NanoSpectralyzer (Applied NanoFluorescence, LLC) using a 10 mm path length quartz cuvette. Fixed excitation wavelengths of 408 and 532 nm lasers were used for acquiring visible and NIR fluorescence spectra of FITC marker and SWCNTs, respectively. The concentration of dispersed SWCNTs in aqueous glycopolymer solutions was calculated using an extinction coefficient value of 0.04163 L mg^−1^ cm^−1^ at 780 nm wavelength (Schöppler et al., 2011). Kinetics of lectin (i.e., PNA and Con A) interaction with Glyco-SWCNTs in PBS buffer solution were monitored using sequence mode data acquisition through time-resolved visible fluorescence spectra of FITC marker at a lectin concentration of 2.23 ± 0.20 μM at room temperature. The corresponding nanotube concentration was kept at 4.21 ± 0.13 μg/ml. Control experiments were performed utilizing DNA-SWCNT dispersions at a nanotube concentration of 6.08 ± 0.52 μg/ml and a Lact-AM 400 homopolymer solution of 0.50 ± 0.01 mg/ml without containing SWCNTs, while keeping the lectin (i.e., PNA and Con A) concentration at 2.23 ± 0.20 µM. These concentrations are roughly similar to that of nanotubes and free, unbound glycopolymers in a typical Glyco-SWCNT sample used for protein interactions.

### Transmission Electron Microscopy

Glyco-SWCNT samples were deposited on a 300-mesh Formvar-carbon lacy TEM grid (glow discharged). 2% (w/w) uranyl acetate was used to negative stain samples on a grid for approximately 1 min. Images were collected using the FEI Tecnai T12 TEM operating at 120 kV with LaB6 filament and imaged using the Gatan 895 UltraScan 4 × 4 k camera. The nanotube concentration of Glyco-SWCNT samples is kept at 13.74 ± 0.50 μg/ml with and without PNA addition at a mass ratio of SWCNTs:PNA = 1:2.

## Results and Discussion

### Noncovalent Complexes of Glyco-SWCNTs

Integrating nanomaterials with biopolymers has been widely explored for applications in biology. Particularly, SWCNTs as a model system of quasi-one-dimensional nanostructures have been utilized for selective interactions with chiral amino acids, carbohydrates, and specific proteins and nucleic acids both *in vitro* and *in vivo* due to their exceptional optical and physiochemical properties and highly tunable surface chemistry (Reuel et al., 2011; Harvey et al., 2019; Santos et al., 2021; Yaari et al., 2021; De los). Helical wrapping of SWCNTs by natural polysaccharides and synthetic glycopolymers is an effective approach to preserve nanotube properties while stabilizing tubes in an aqueous environment (Star et al., 2002; Hasegawa et al., 2004; Dohi et al., 2006; Cantwell et al., 2020). In addition to van der Waals attractions and hydrophobic interactions between the hydrophobic faces of the pyranose rings and the hydrophobic surface of nanotubes, intra- and inter-molecular hydrogen bonds of carbohydrates with neighboring sugar units and water molecules further stabilize the helical conformation of polymers on the surface of nanotubes (Çarçabal et al., 2005; Bristol et al., 2020; Liu et al., 2012).

**FIGURE 1:**
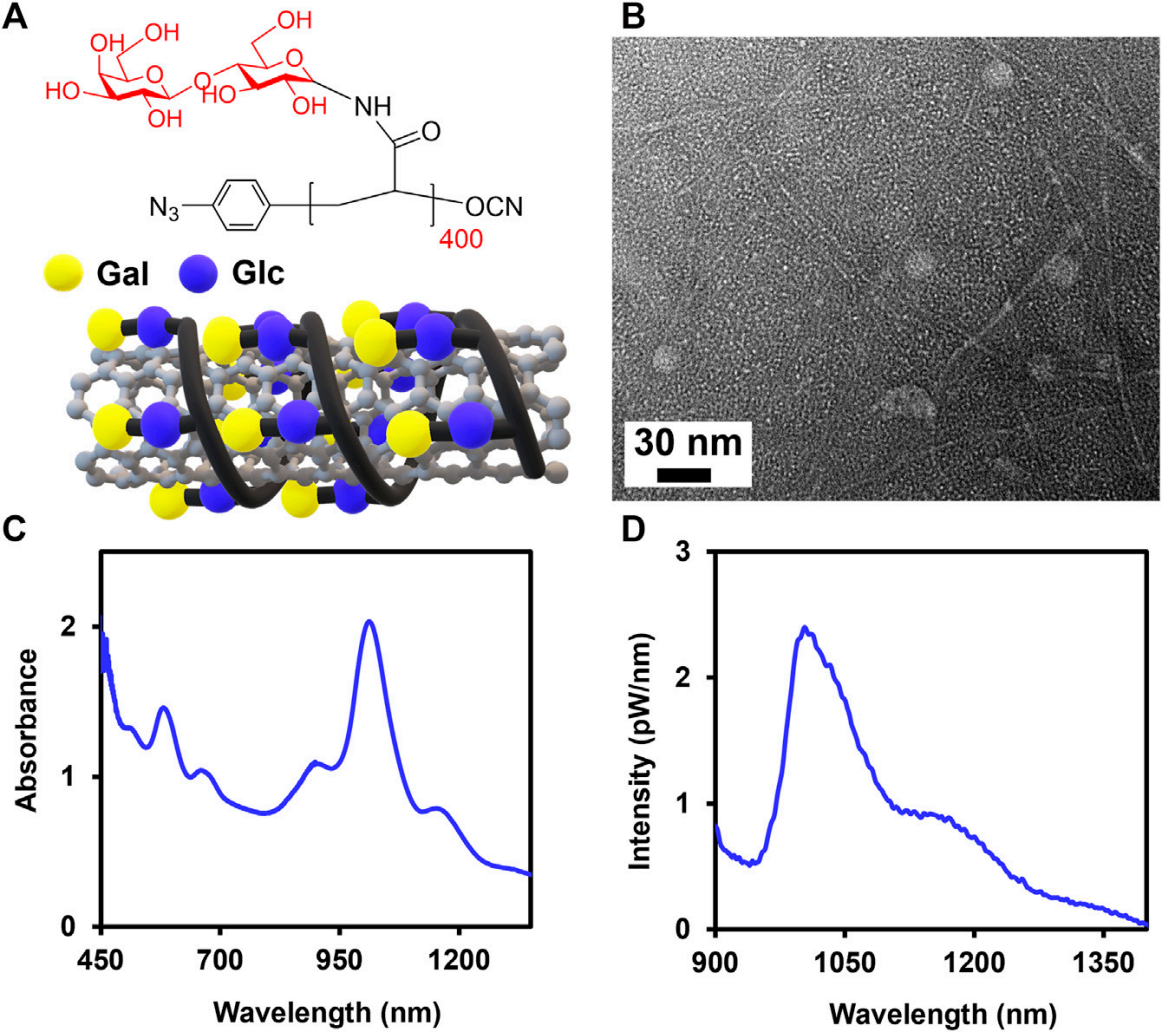
Noncovalent complexation of SWCNTs with Lact-homopolymer. **(A)** Chemical structure of Lact-AM 400 homopolymer and the schematic of a SWCNT wrapped by Lact-homopolymer. **(B)** TEM of Glyco-SWCNT sample containing excess, unbound Lact-homopolymer. Representative **(C)** absorbance and **(D)** NIR fluorescence spectra of supernatant samples of 0.1 mg/ml SWCNTs dispersed in an aqueous solution of 0.4 mg/ml Lact-AM 400. Nanotube samples were diluted by a factor of 2× in DI water for optical characterization.

In this study, we complexed SWCNTs noncovalently with Lact-homopolymers synthesized from lactosylacrylamide (i.e., Lact-AM) monomer with long polymer chain lengths of *n* = 400 and 415 (i.e., Lact-AM 400 and Lact-AM 415) (Figure 1 and Supplementary Figure S1) (Cantwell et al., 2020). We have reported previously that homopolymers synthesized from disaccharide *ß*-lactose, which consists of a terminal *ß*-galactose (Gal) and an internal *ß*-glucose (Glc) groups, with long chain lengths (i.e., roughly n > 85) produce stable glycopolymer-wrapped SWCNT complexes in water (Figure 1A). In our current work, we further optimized the dispersion condition of SWCNTs in water using Lact-AM 415 homopolymer. The optimum ultrasonication time was determined to be a total of 90 min with 30 min rest between each sonication period of 45 min, obtaining a nanotube dispersion yield of roughly 42%, as compared to the previously reported value of ≈38%, in supernatant samples (Supplementary Figure S2 and Supplementary Table S1) (Cantwell et al., 2020). The 30 min rest between each sonication steps is necessary to prevent overheating of Glyco-SWCNT samples, which may cause polymer degradation and a decreased dispersion quality of nanotubes. We also confirmed the optimum mass ratio of SWCNTs:glycopolymer = 1:4, which was utilized in our previous work (Cantwell et al., 2020), in stabilizing the complexes using the new sonication time (Supplementary Figure S3). Particularly, the dispersion quality and yield of SWCNTs improve continuously as the SWCNTs:Lact-AM 415 mass ratio changes from 1:0.5, which did not result in nanotube dispersion, to 1:4, as evidenced by the increasing intensity and sharpening of characteristics optical transition peaks of SWCNTs in the NIR wavelength region (Supplementary Figure S3). Further increasing the glycopolymer content to a mass ratio of SWCNTs:Lact-AM 415 = 1:8 did not improve the nanotube dispersion (Supplementary Figure S3). The optimized dispersion condition of SWCNTs produces individually dispersed nanotubes coated by glycopolymers as evidenced by transmission electron microscopy (TEM) and optical spectroscopy characterization (Figure 1). A few globular structures shown in TEM are the unbound glycopolymers in the sample (Figure 1B). Additionally, E_11_ and E_22_ transition peak features of SWCNTs are clearly shown in the Glyco-SWCNT absorption, where the maximum E_11_ absorbance peak near 1013 nm wavelength corresponds to that of glycopolymer-coated (6,5) SWCNT species which is abundant in the synthetic nanotube material used in this work (Figure 1C). Moreover, Glyco-SWCNT complexes exhibit the NIR fluorescence feature that is intrinsic to semiconducting nanotubes (Figure 1D). For the remainder of the work, we stabilized SWCNTs using the Lact-AM 400 homopolymer at the optimum condition of SWCNTs:polymer = 1:4 mass ratio and the sonication time mentioned above and studied the interactions of Glyco-SWCNT complexes with various proteins.

### Interaction of Glyco-SWCNTs With Proteins at Varying Concentrations

We selected various lectins to interact with Glyco-SWCNTs including a homotetrameric PNA with specific affinity to *ß*-Gal, a tetrameric Con A with specific binding to *a*-Glc and *a*-mannose (i.e., Man at pH > 7), and a tetrameric GNA which is highly specific for *a*-Man (Dam and Brewer 2002). Tetrameric lectins with multiple sugar-binding sites further promote interactions with a multivalent ligand delivery system. It is noteworthy to mention that, although the results varied among different carbohydrate-containing systems and characterization methods, Con A has been shown to bind specifically to the *ß*-Glc pendant groups of linear and branched glycopolymers both in their extended chain conformation and assembled micelle structures (Pasparakis et al., 2007; Chen et al., 2010). We also utilized bovine serum albumin (BSA) that is non-specific for carbohydrate interaction as a control.

**FIGURE 2:**
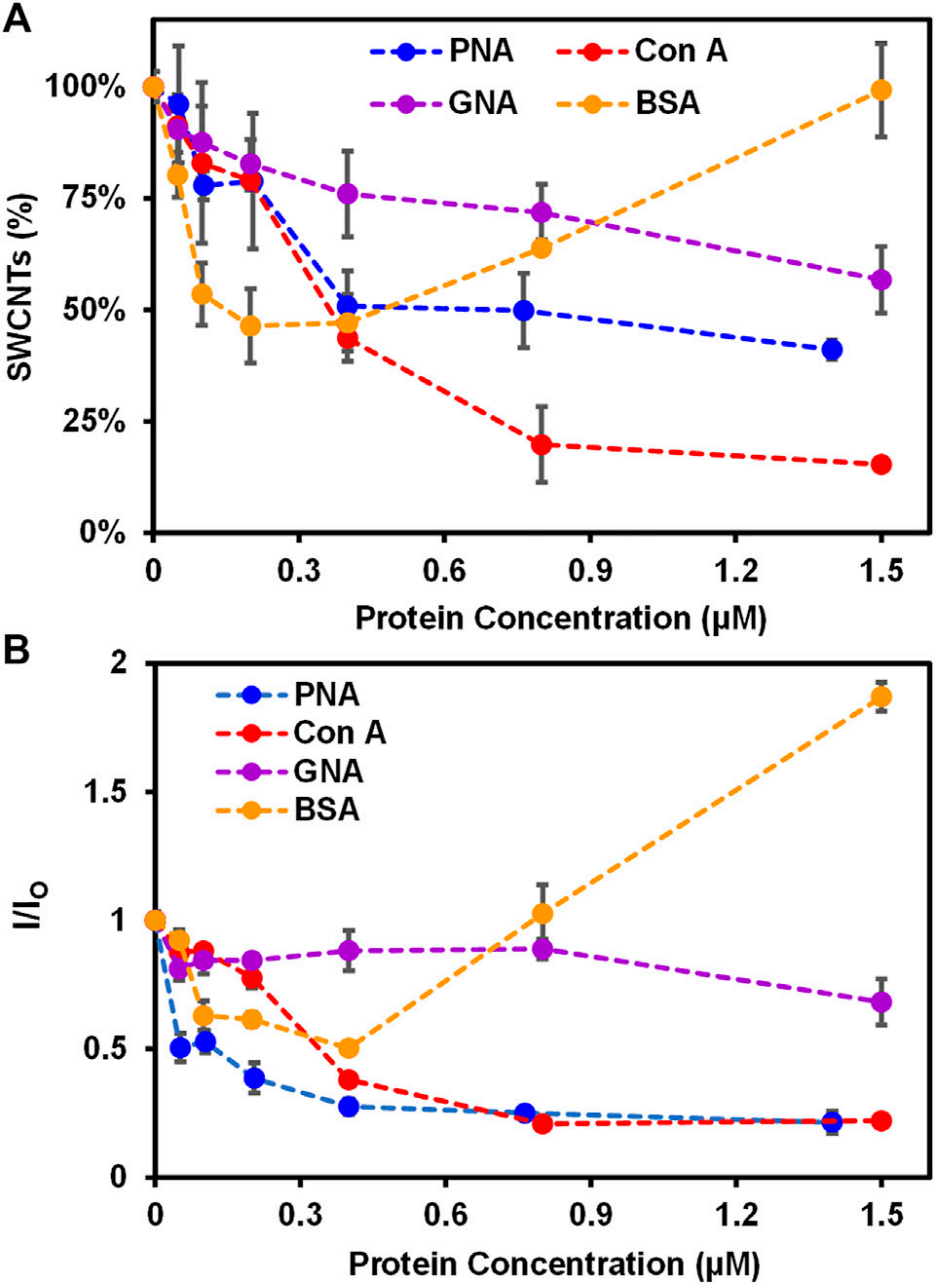
Interactions of Glyco-SWCNTs with proteins. **(A)** Percentage of SWCNTs remaining in the supernatant determined from the sample absorption at 780 nm and the corresponding changes in **(B)** the NIR emission intensity ratio at the maximum E_11_ peak wavelength (i.e., 1013 nm) of Glyco-SWCNTs after incubating nanotubes with different proteins including PNA, Con A, GNA, and BSA, respectively, for 5 min. The nanotube concentration of Glyco-SWCNTs is kept constant at 4.21 ± 0.13 μg/ml, while the protein concentration ranges from 0 to 1.50 ± 0.20 µM. A fixed excitation wavelength of 532 nm laser was used for NIR fluorescence spectra measurements.

Specifically, various proteins of increasing concentrations (i.e., 0—1.5 ± 0.20 µM) were incubated with Glyco-SWCNTs at a constant nanotube concentration of 4.21 ± 0.13 μg/ml in PBS buffer solution for 5 min to measure the SWCNT absorption and NIR fluorescence (Figure 2; Supplementary Figures S4, S5). The shorter time period of 5 min was selected after incubating PNA-FITC and Glyco-SWCNTs for 5 and 10 min, respectively, where the visible fluorescence of FITC marker quenched after both incubation time periods (Supplementary Figure S6). The binding of a FITC-labeled lectin to a receptor carbohydrate ligand generally causes the quenching of FITC fluorescence due to the fluorophore conjugated lectin undergoing conformational changes and multivalent interaction as well (Breen et al., 2016). The aromatic FITC molecule is also known to adsorb onto the SWCNT surface through hydrophobic interactions, leading to the quenching of its fluorescence (Zhang et al., 2013). The carbohydrate-mediated interaction between the terminal Gal group of Lact-homopolymers and PNA-FITC may drive FITC into close proximity to Glyco-SWCNTs and the potential adsorption of FITC onto the nanotube surface may cause further quenching of FITC fluorescence. As expected, we observed a continuous and drastic decrease of dispersed nanotubes to ≈51% of the initial SWCNT mass content of Glyco-SWCNTs, based on the absorption measurements, with increasing PNA concentration of up to 0.40 ± 0.01 µM due to the formation of crosslinked aggregates of nanotubes (Figure 2A and Supplementary Figure S4). Multivalent interactions of carbohydrate ligands and lectins are known to form the crosslinked network in biology and the crosslinking of lectins with glyconanomaterials have been observed previously as well (Brewer 2001; Liyanage and Yan 2020). Further increase in the PNA concentration up to 1.40 ± 0.20 µM led to a slight decrease in the dispersed nanotube percentage to ≈41%. This indicates that a saturated level of interactions between Glyco-SWCNTs and PNA occurs around 0.40 ± 0.01 µM of lectin at the given initial SWCNT concentration of 4.21 ± 0.13 μg/ml, which roughly corresponds to a Glyco-SWCNTs:PNA mass ratio of 1:11.

Interestingly, when Con A was incubated with Glyco-SWCNTs, we observed more drastic aggregation of nanotubes with increasing lectin concentration, resulting in ≈20% nanotubes remaining in the supernatant sample at 0.80 ± 0.01 µM of lectin (Figure 2A). With further increase in the Con A concentration to 1.50 ± 0.20 µM, no obvious change was observed for the dispersed nanotubes remaining in the supernatant (i.e., ≈15% SWCNTs). This suggests that the interaction between Glyco-SWCNTs and Con A saturated near 0.80 ± 0.01 µM of lectin (i.e., a mass ratio of Glyco-SWCNTs:Con A ≈ 1:19). We speculate that the wrapping conformation of polymers around nanotubes leads to the unique nanostructure-induced interaction of the exposed, internal Glc groups of Lact-homopolymers with Glc-binding Con A. This could be due to non-specific interaction between Con A and Glyco-SWCNTs as well, which requires further investigation in future work. Additionally, the increased aggregation of nanotubes when incubated with Con A suggests that the Glc unit plays an important role in stabilizing the wrapping conformation of Glyco-SWCNTs through hydrophobic interaction between the pyranose rings (i.e., the Glc unit) and the nanotube surface.

In fact, we observed previously that glycopolymers synthesized from monosaccharide Glc can stabilize SWCNTs in water to a certain extent, while monosaccharides Gal-containing polymers did not disperse nanotubes (Cantwell et al., 2020). The potential Con A binding to Glc units competes with the stabilizing interaction between Glc rings with SWCNTs, and the removal of Glc units from the nanotube surface leads to the conformational change of wrapped polymers, causing the increased aggregation of nanotubes in water. This observation also suggests that the noncovalent system of Glyco-SWCNTs provides an advantage of studying the carbohydrate-protein interactions with improved sensitivity *via* the competitive binding of molecules, as a biologically active ligand-receptor pair may act as a competitor to displace the lower affinity glycopolymer-SWCNT interaction (Antonik et al., 2016). Furthermore, the cooperative hydrogen-bond networks created by the intra- and inter-molecular hydrogen bonds with the hydroxyl groups of neighboring carbohydrates and the environment, such as water molecules, play a major role in stabilizing hydrated carbohydrate conformation as well as molecular recognition (Hawley et al., 2002; Çarçabal et al., 2005). The strongly rigidified conformation of disaccharide Lact-homopolymers around the SWCNT surface may create a specific solvated state of the internal Glc groups with certain conformation that can potentially involve in the cooperative hydrogen bond recognition of Con A. Although studies on the carbohydrate-protein interaction so far have been mainly focused on the interaction behavior of the terminal glycans with lectins, the binding of lectins to the internal carbohydrate units of polysaccharides and glycopeptides has been reported previously (Brewer and Bhattacharyya 1986; Dam et al., 1998; Meagher et al., 2005; Wu et al., 2016). Our future work will focus on further understanding the possible role of internal sugar units on interacting with lectins, which will broaden our knowledge about the binding profile of lectins as well as enable the development of versatile glyconanomaterials to detect carbohydrate-protein interactions with improved selectivity and sensitivity.

As for the Man-binding GNA, we observed a slow decrease in the percentage of nanotubes retained in the Glyco-SWCNT supernatants with increasing lectin concentration and obtained the minimum aggregation of nanotubes with ≈57% SWCNTs remaining at 1.50 ± 0.20 μM of lectin as compared to PNA and Con A (Figure 2A). This is expected as Lact-homopolymers do not possess carbohydrate units that have specific binding to GNA. Additionally, non-carbohydrate binding BSA showed a different interaction behavior with Glyco-SWCNTs as compared to that of lectins (Figure 2A). At lower BSA concentrations of up to 0.20 ± 0.01 μM, the SWCNT percentage in the supernatants decreased continuously to ≈46% with increasing protein concentration possibly due to the electrostatic interactions between BSA and dispersed SWCNTs, causing the destabilization of Glyco-SWCNT complexes and nanotube aggregation (Sun et al., 2018). However, at higher BSA concentrations above 0.40 ± 0.01 μM, the uniform adsorption of BSA onto the surface of tubes *via p*-π stacking and hydrophobic interactions with hydrophobic aromatic residues of BSA (i.e., Trp, Phe, Tyr) facilitates the stabilization of SWCNTs (Sun et al., 2018; Ge et al., 2011). Especially at 1.50 ± 0.20 μM of BSA, the SWCNT content in the supernatant sample remained the same without forming nanotube aggregates.

The corresponding NIR fluorescence measurements of SWCNTs revealed the similar interaction behavior of proteins with Glyco-SWCNTs. The photoluminescence of nanotubes is highly sensitive to small perturbations in the external environment than its absorption, allowing rapid measurements through monitoring spectral changes of SWCNT emission peaks in the NIR (Xhyliu and Ao 2020; Weisman and Bachilo 2003). Figure 2B shows the emission intensity ratio *I/I*_*O*_ of nanotubes at the maximum E_11_ peak wavelength near 1002 nm corresponding to that of (6,5) SWCNTs, where $I_{O}$ and I are the magnitude of the maximum E_11_ emission peaks before and after protein incubation. The representative NIR fluorescence spectra of Glyco-SWCNTs that are incubated with various proteins at different concentrations are shown in Supplementary Figure S5. The removal of nanotubes from the supernatants due to the formation of crosslinked aggregates results in the quenched fluorescence of SWCNTs, which is affected by the concentration and the surface coverage of SWCNTs as well as the aggregated state of SWCNTs (Landry et al., 2015; Xhyliu and Ao 2020). Specifically, the emission intensity ratio of SWCNTs decreased slightly to 0.68 ± 0.13 with increasing concentration of lectin GNA up to 1.50 ± 0.20 μM. However, it dropped significantly with increasing lectin concentration for lectins PNA and Con A, and reached a plateau of *I/I*_*O*_ values of roughly 0.27 ± 0.04 and 0.21 ± 0.02, respectively, near 0.40 ± 0.01 μM of lecin PNA and 0.80 ± 0.01 μM of lecin Con A. With BSA incubation, the emission intensity ratio of SWCNTs initially decreased to 0.50 ± 0.02 with increasing protein concentration up to 0.40 ± 0.03 μM, followed by a drastic increase at high BSA concentrations up to 1.50 ± 0.20 μM.

### Competitive and Sequential Binding of Lectins With Glyco-SWCNTs

**FIGURE 3:**
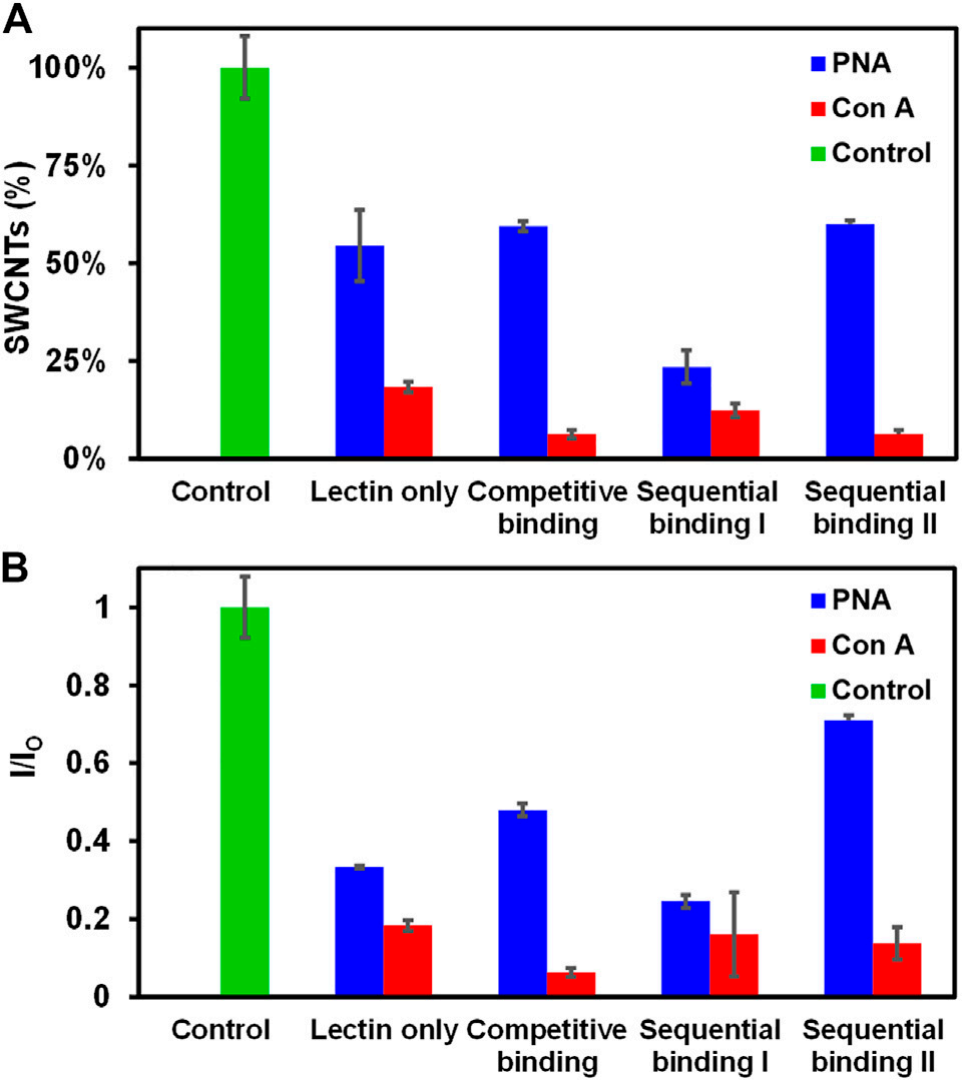
Comparison of different methods of lectin interactions with Glyco-SWCNTs. **(A)** Percentage of SWCNTs remaining in the supernatant determined from the sample absorption at 780 nm and the corresponding changes in **(B)** the NIR emission intensity ratio at the maximum E_11_ peak wavelength (i.e., 1013 nm) of Glyco-SWCNTs after incubation with lectins and free sugar *ß*-lactose. The control was performed by measuring the optical spectra of Glyco-SWCNTs only incubated in PBS buffer solution for 5 min without adding lectins and free sugar *ß*-lactose. The sample concentrations are 4.21 ± 0.13 μg/ml nanotubes for Glyco-SWCNTs, 0.77 ± 0.01 µM lectin (i.e., PNA and Con A, respectively), and 0.36 ± 0.05 mg/ml free sugar *ß*-lactose, respectively. A fixed excitation wavelength of 532 nm laser was used for NIR fluorescence spectra measurements.

We further investigated the binding behavior of PNA and Con A with Glyco-SWCNTs using different binding methods including lectin only (i.e., without free lactose added), competitive binding (i.e., incubation of Glyco-SWCNTs, lectin, and free lactose simultaneously), sequential binding I (i.e., incubation of Glyco-SWCNTs and lectin first, then adding free lactose), and sequential binding II (i.e., incubation of lectin with free lactose first, then adding Glyco-SWCNTs). We used the lectin concentration of 0.77 ± 0.01 µM, where the SWCNT percentage remaining in the supernatants is within the plateau range for the selected nanotube concentration (i.e., 4.21 ± 0.13 μg/ml) (Figure 2A). The visible fluorescence measurements after incubating lectins (i.e., PNA and Con A, respectively) with Glyco-SWCNTs showed quenched FITC fluorescence for different binding methods, indicating that almost all lectins interact with Glyco-SWCNT complexes at the given concentrations of lectins and SWCNTs (Supplementary Figure S7). Figure 3A shows the percentage of SWCNTs remaining in the supernatant samples obtained from the SWCNT absorption measurements after incubating with lectins. The amount of free sugar *ß*-lactose used in competitive and sequential binding tests roughly corresponds to the sugar content of Lact-homopolymers, including the nanotube-bound and excess unbound polymers in Glyco-SWCNT samples. The control test confirmed the stability of Glyco-SWCNTs only in PBS buffer solution during the incubation period tested in our experiments. When incubating Glyco-SWCNTs with PNA, the sequential binding I resulted in the highest amount of nanotube aggregation, leaving ≈23% of SWCNTs remaining in supernatants compared to roughly 54—60% nanotubes retained for the rest of the binding methods (i.e., lectin only, competitive binding, and sequential binding II) (Figure 3A). For the competitive and sequential binding II of PNA, similar percentage of SWCNTs retained in supernatants (i.e., ≈60%), indicating that the lectin interacts more strongly with Glyco-SWCNTs than with free sugar *ß*-lactose. For the sequential binding I, the total incubation time of SWCNTs with lectins is 10 min compared to the 5 min incubation time used for other binding methods. However, a comparative study of incubating PNA only with SWCNTs for 5 and 10 min resulted in the same percentage of nanotubes remaining in supernatants (i.e., ≈55%), indicating that the longer incubation time is not likely the reason for causing the greater aggregation of nanotubes obtained for sequential binding I (Supplementary Figure S8). Instead, we speculate that for the sequential binding I, PNA initially interacts with Glyco-SWCNTs to form crosslinked aggregates. The addition of free sugar *ß*-lactose afterwards may further promote the formation of larger and tighter crosslinked networks of Glyco-SWCNTs due to the interstitial binding of free sugar with available binding sites of PNA that are already bound on the nanotube surface, which will be examined further in our future work.

Compared to the terminal Gal-binding PNA, the Con A binding of Glyco-SWCNTs induced greater aggregation of nanotubes, resulting in roughly 6—18% of SWCNTs remaining in the supernatants for different lectin binding methods (Figure 3A). This is consistent with our previous observation that the potential binding of Con A to Glc unit destabilizes the wrapping conformation of Glyco-SWCNT complexes by peeling off the polymers from the nanotube surface. In addition to the formation of the crosslinked lattice structures, the increased aggregation of nanotubes is promoted by the conformational change of wrapped polymers upon Con A binding. The interaction behavior of Glyco-SWCNTs and Con A is not obviously affected with the addition of free sugar *ß*-lactose, suggesting that the internal Glc group of the free sugar is not readily accessible for Con A binding. However, the wrapping of glycopolymers around SWCNTs likely leads to the enhanced avidity of Con A with Lact-containing polymer on SWCNTs. Therefore, the differences in the percentage of SWCNTs remaining in the supernatants among different lectin binding methods are smaller for Con A as compared to PNA.

Additionally, the NIR fluorescence measurements of nanotubes showed the similar interaction behavior of lectins with Glyco-SWCNTs as deduced from the absorption measurements (Figure 3B). For the incubation with PNA, the emission intensity ratio *I/I*_*O*_ of nanotubes obtained at the maximum E_11_ peak wavelength of 1002 nm showed the minimum value of 0.25 ± 0.02 for the sequential binding I, resulting in the maximum removal of nanotube aggregates. The highest *I/I*_*O*_ value is obtained for the sequential binding II of PNA, which is potentially caused by the decreased amount of lectin binding sites available for interacting with Glyco-SWCNTs due to the possible interaction of PNA with free sugar *ß*-lactose during the initial incubation step. This decreased level of nanotube aggregation is perhaps more sensitively measured by SWCNT photoluminescence compared to the nanotube absorption. The *I/I*_*O*_ value for the competitive binding of PNA (i.e., 0.48 ± 0.02) is slightly higher than that of lectin only (i.e., 0.33 ± 0.01), indicating that the existence of free sugar *ß*-lactose may interfere with the interaction between PNA and Glyco-SWCNTs to a certain degree, leading to a lesser degree of nanotube aggregation. As for the incubation of Glyco-SWCNTs with Con A, the emission intensity ratio of nanotubes ranges from 0.06 ± 0.01 to 0.18 ± 0.01 for different lectin binding methods, which are much smaller than those obtained for PNA. These observations provide additional evidence that Con A and PNA have different binding behaviors when interacting with Glyco-SWCNTs. Specifically, the potential binding of Con A to the Glc groups de-stabilize the wrapping conformation of polymers around nanotubes, causing greater nanotube aggregations. Whereas, the interaction of PNA with Glyco-SWCNTs, mediated by the terminal Gal groups, leads to the known phenomenon of forming cross-linked aggregates.

### Kinetics of Lectin Interaction With Glyco-SWCNTs

**FIGURE 4:**
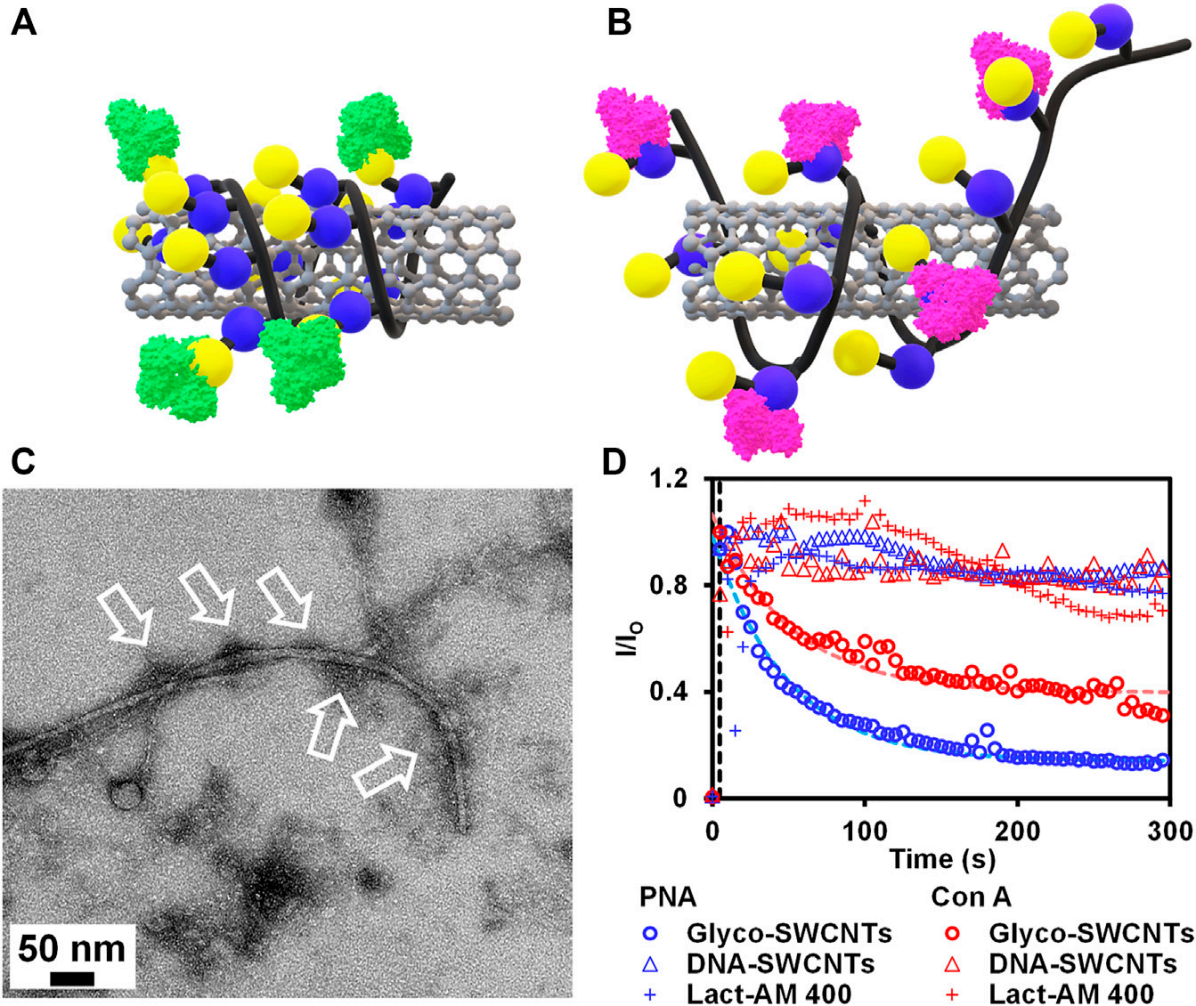
Schematics depicting the binding of **(A)** PNA to the external Gal group and **(B)** Con A to the internal Glc group of Lact-homopolymer that is wrapped around the SWCNT. **(C)** TEM of Glyco-SWCNT sample at a nanotube concentration of 13.74 ± 0.50 μg/ml after incubating with 26.00 ± 0.05 μg/ml PNA. **(D)** Kinetics of lectin (i.e., PNA and Con A, respectively) interaction with Glyco-SWCNTs, DNA-SWCNTs, and Lact-AM 400 polymer, respectively, that are measured from the emission intensity ratio change of lectin FITC marker at 525 nm peak wavelength as a function of time. A fixed excitation wavelength of 408 nm laser was used for visible fluorescence spectra measurements.

As discussed previously, we propose a different interaction behavior between PNA and Con A with Glyco-SWCNTs as shown in Figures 4A,B. Specifically, PNA binds to the terminal Gal unit of Lact-homopolymer that are wrapped around nanotubes, while the binding of Con A to the internal Glc unit overcomes the hydrophobic interaction between the Glc rings and the nanotube surface. This greatly disrupts the polymer wrapping conformation and may peel off the polymer coatings from the nanotube surface, leading to increased nanotube aggregations in water. The TEM of Glyco-SWCNTs incubated with PNA shows dark regions near nanotubes, that are indicated by arrows, likely revealing the binding of lectins with carbohydrate ligands coated on the surface of nanotubes (Figure 4C). These dark regions were not observed for individually dispersed Glyco-SWCNT samples (Figure 1B).

To further reveal the different interaction behaviors of PNA and Con A with Glyco-SWCNTs, we measured the kinetics of carbohydrate-lectin interactions by monitoring the change in the visible emission intensity of lectin FITC markers. Figure 4D plots the time dependence of visible emission intensity ratio *I/I*_*O*_ of FITC at the peak wavelength of 525 nm, where $I_{O}$ and I are the magnitude of emission peaks corresponding to lectin FITC before and after adding the interacting species (i.e., Glyco-SWCNTs, DNA-SWCNTs, and Lact-AM 400). The sequence-controlled DNA utilized in this work is known to form an ordered wrapping structure around nanotubes *via* noncovalent complexation, stabilizing DNA-SWCNT complexes in water through electrostatic interactions due to the charge-carrying phosphate−sugar DNA backbone (Ao et al., 2016; Roxbury et al., 2012). An excess amount of lectins (i.e., 2.23 ± 0.20 μM) were utilized to interact with Glyco-SWCNTs containing 4.21 ± 0.13 μg/ml of nanotubes to measure the fluorescence intensity of lectin FITC during the total reaction time of 5 min. The nanotube and polymer concentrations of DNA-SWCNTs and Lact-AM 400 control samples roughly correspond to the SWCNTs and free, unbound glycopolymers, respectively, of the Glyco-SWCNT samples. The time traces of decrease in intensity ratio of lectin FITC when interacting with Glyco-SWCNTs can be modeled using single exponential fit (Supplementary Figure S9 and Supplementary Table S2, *R* ^*2*^ > 0.9 for all fits from three repeats), suggesting that the carbohydrate-mediated interaction of lectins operates as a pseudo-first order reaction (Roxbury et al., 2011; Jena et al., 2017; Xhyliu and Ao 2020). The time constant t deduced from the spectral fit corresponds to the inverse rate constant 1/k , where a longer time corresponds to a stronger binding affinity of the wrapping Lact-homopolymer to SWCNTs (Xhyliu and Ao 2020).

For Con A interacting with Glyco-SWCNTs, we obtained a deduced time constant of ≈66 s, which is roughly 73% larger than that of PNA (i.e., ≈38 s) (Supplementary Table S2). This suggests that the internal Glc unit of Lact-homopolymers binds more strongly to the SWCNT surface than the external Gal unit, leading to a slower competitive binding between the recognition pairs of Con A and Glc units as compared to that of PNA and Gal units. The potential binding of Con A to the internal Glc units displaces the stabilizing interaction between the Glc units and SWCNTs, leading to a greater level of nanotube aggregation as discussed previously. In comparison, the emission intensity ratio of FITC marker of both lectins (i.e., PNA and Con A) obtained from the control samples of DNA-SWCNTs and Lact-AM 400 did not show obvious changes as a function of time (Figure 4D). This suggests that the interaction between lectins and Glyco-SWCNTs is primarily mediated by the carbohydrates displayed on the surface of nanotubes, as nanotubes with DNA wrapping on the surface did not interact with lectins. The rigid wrapping conformation of glycopolymers enabled by complexing with SWCNTs is necessary to significantly promote the multivalent glyconanomaterial-protein interactions, as free Lact-AM 400 alone did not result in obvious interactions with lectins. These observations suggest that the possible non-specific interaction of Con A with the partially exposed surface of nanotubes that are stabilized through polymer wrapping is unlikely. More importantly, our results demonstrate that the wrapping of glycopolymers around nanotubes potentially enhances the accessibility and avidity of the Con A binding to the Glc unit of Lact-homopolymers, enabling the previously unachieved interaction between Con A and the Glc unit of lactose-conjugates hosted on a substrate (i.e., SWCNTs in this work). Further investigations of the Con A specific or non-specific interactions of Glyco-SWCNTs are worthy of future studies. It is also worth pointing out that utilizing SWCNTs complexed with glycopolymers containing disaccharide groups other than lactose as well as trisaccharide groups may provide additional details on the unique interaction behavior of lectins with internal sugar units, which requires future experiments. Combined, our work provides an important insight on the unique interactions of multivalent carbohydrate ligands and lectins, that are enabled by the integration of nanomaterials with glycopolymers.

## Conclusion

In summary, we have demonstrated distinct interactions of carbohydrates and specific lectins utilizing noncovalent complexes of SWCNTs wrapped by glycopolymers. Gal-specific lectin PNA binding to the terminal Gal of Lact-polymers on the surface of SWCNTs was confirmed by optical spectroscopy and SEM characterization as well as kinetics of lectin interaction of Glyco-SWCNTs. In addition, an unexpected interaction between Con A and the Lact-homopolymer-complexed SWCNTs was observed. We speculate that the rigid, wrapping conformation of glycopolymers formed on the nanotube scaffold enables the potential interaction between Con A and the Glc of Lact-homopolymers. The increased aggregation behavior of nanotubes observed for Con A interaction as well as the kinetics of carbohydrate-mediated interaction of lectins with Glyco-SWCNTs revealed the key role of the Glc groups in stabilizing the wrapping structure of glycopolymers around the nanotubes through hydrophobic interactions. Our findings provide insights for designing novel glyconanomaterials with unique optical and carbohydrate functionalities for profiling a broad range of carbohydrate-lectin interactions with enhanced specificity and sensitivity. This work will also contribute to the development of potential biomedical applications of glyconanomaterials, including biosensors, drug delivery, and pathogen inhibition.

## Data Availability

The original contributions presented in the study are included in the article/Supplementary Material, further inquiries can be directed to the corresponding authors.
