# Supplementary material for: Glycopolymer-Wrapped Carbon Nanotubes Show Distinct Interaction of Carbohydrates With Lectins
Source: Front Chem. 2022 Mar 3;10:852988. doi: 10.3389/fchem.2022.852988 (PMC8927622; doi:10.3389/fchem.2022.852988)
Supplement: Supplementary file 1 [file Presentation1.pdf]

Supplementary Material for

Glycopolymer-Wrapped Carbon Nanotubes Show Distinct Interaction of Carbohydrates with Lectins

Ana M. DiLillo,[1] *Ka Keung Chan,*[2] *Xue-Long Sun,*[1,2,]* *Geyou Ao*[1,]*

[1]Department of Chemical and Biomedical Engineering, Washkewicz College of Engineering, Cleveland State University, 2121 Euclid Avenue, Cleveland, OH 44115, USA

[2]Department of Chemistry, Center for Gene Regulation in Health and Disease (GRHD), Cleveland State University, 2121 Euclid Avenue, Cleveland, OH 44115, USA

***Correspondence**

Geyou Ao: g.ao@csuohio.edu

Xue-Long Sun: x.sun55@csuohio.edu

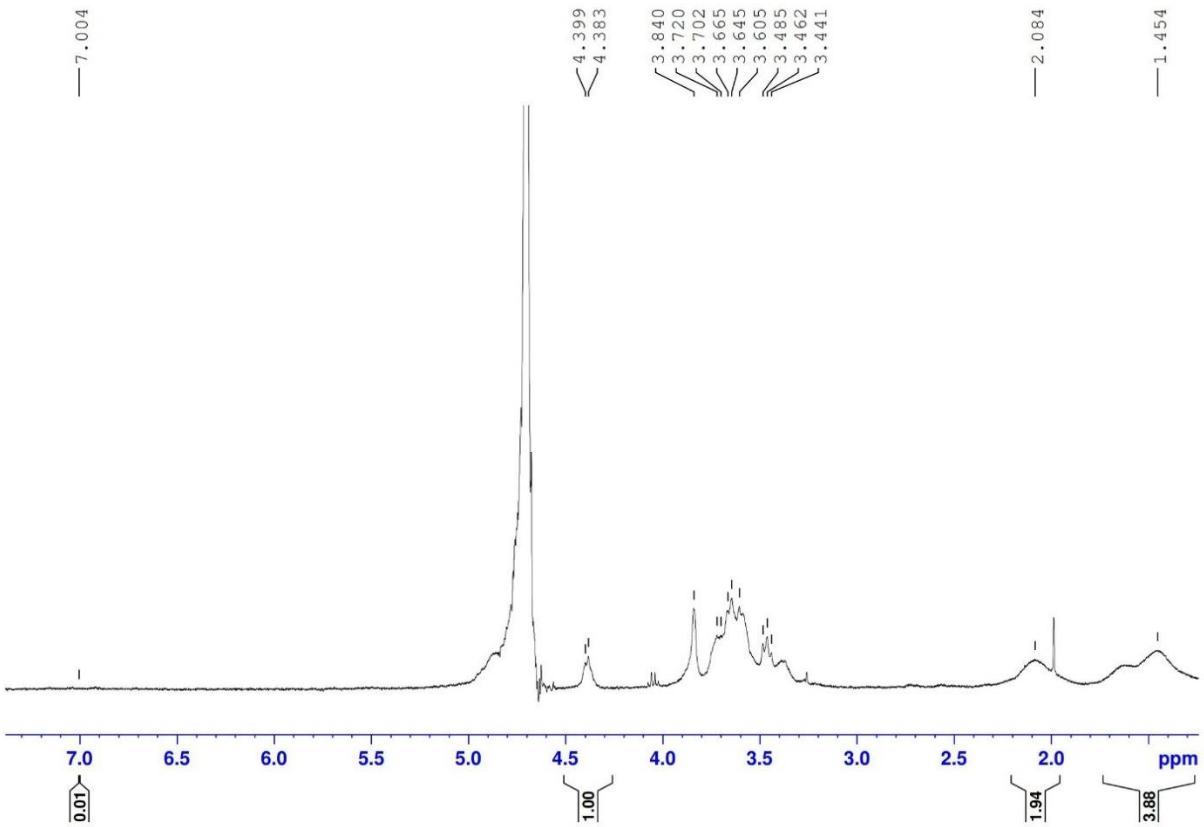

Figure S1. ^{1}H NMR of Lact-AM 400 homopolymer (in D_2O, 400 MHz NMR).

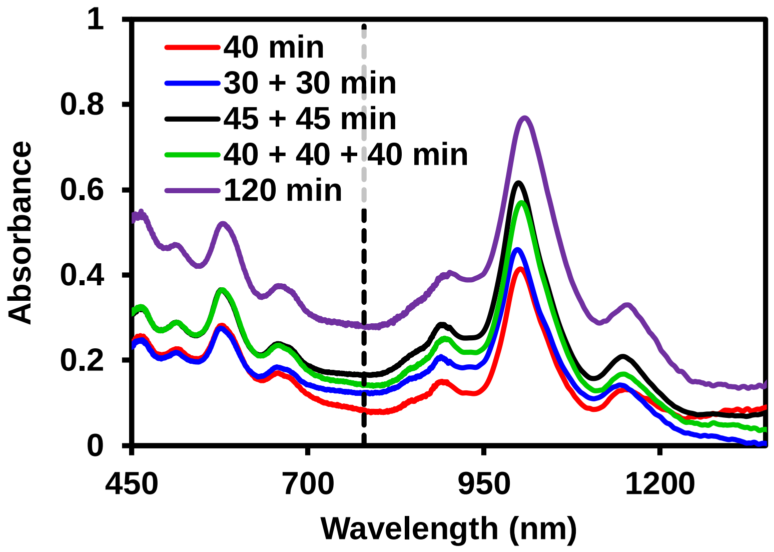

Figure S2. Absorbance spectra of 0.1 mg/mL SWCNTs dispersed in aqueous solutions of 0.4 mg/mL Lact-AM 415 glycopolymer by tip sonication at various time periods of 40, 30 + 30, 45 + 45, 40 + 40 + 40, and 120 min. The plus sign indicates a 30-min rest between each sonication step. Supernatant samples were diluted by a factor of 10× in DI water for optical characterization, except the sample with 120 min sonication which was not diluted.

Table S1. Dispersion yield of 0.10 mg/mL SWCNTs stabilized in aqueous solutions of 0.40 mg/mL Lact-AM 415 glycopolymer by tip sonication at various time periods. The plus sign indicates a 30-min rest between each sonication step. The SWCNT concentrations in supernatant samples were determined using the extinction coefficient value of 0.04163 L/mg·cm at 780 nm (Schöppler et al. 2011).

Sonication time (min)	SWCNT concentration (µg/mL)	SWCNT dispersion yield (%)
40	19.7	19.7
30 + 30	29.6	29.6
45 + 45	42.1	42.1
40 + 40 + 40	34.2	34.2
120	6.7	6.7

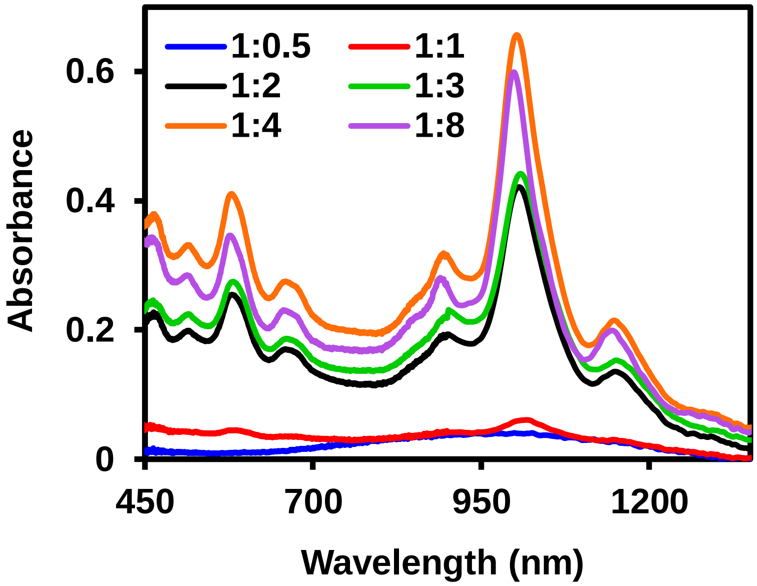

Figure S3. Absorbance spectra of 0.10 mg/mL SWCNTs dispersed in aqueous solutions of Lact-AM 415 glycopolymer at different SWCNTs:Lact-AM 415 mass ratios of 1:n, where n is 0.5, 1, 2, 3, 4, and 8, respectively. All samples were prepared by tip sonication for 45 + 45 minutes with 30 min rest between each sonication step. All supernatant samples were diluted by a factor of 10× in DI water for optical characterization.

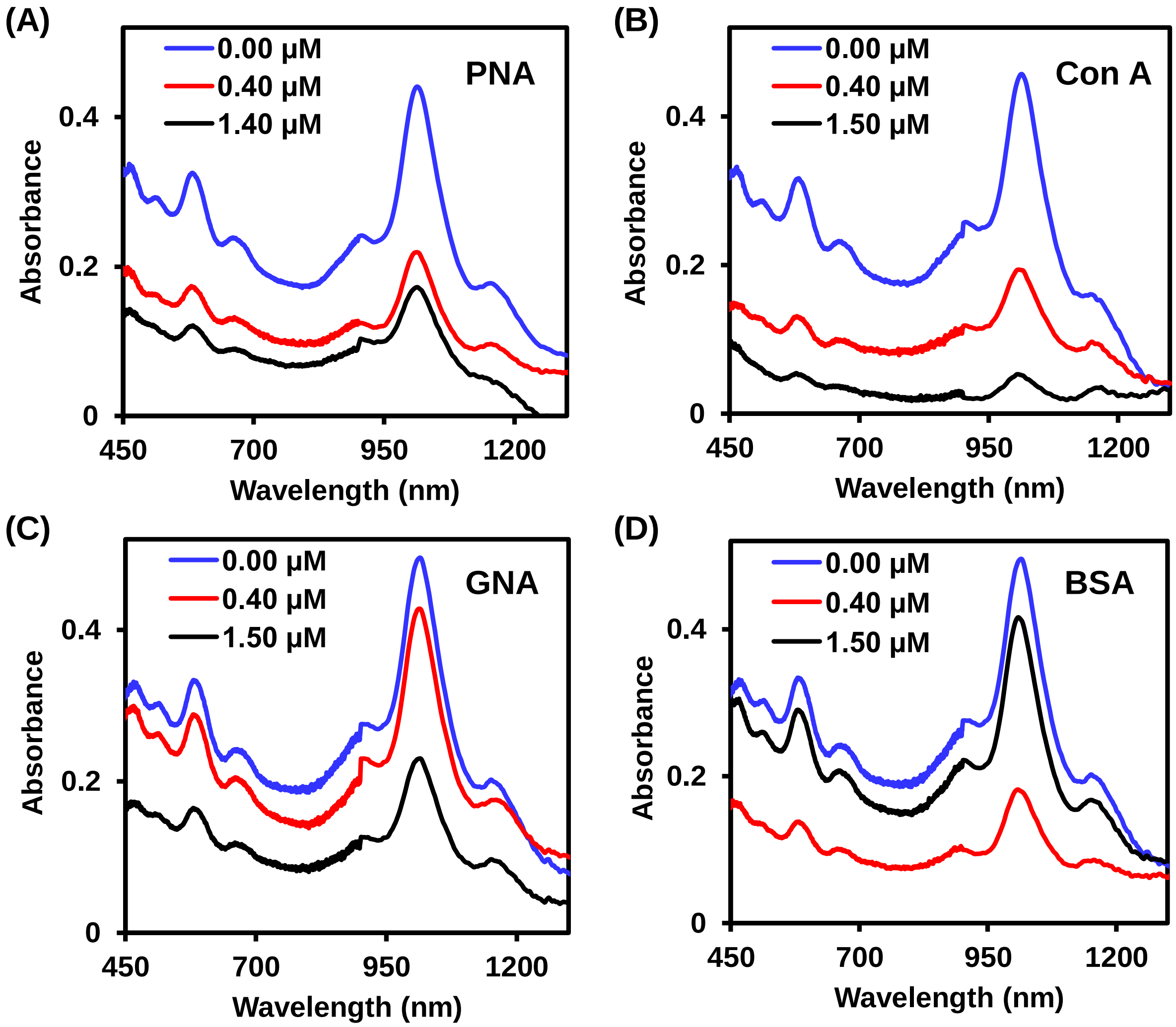

Figure S4. Representative absorbance spectra of Glyco-SWCNTs (i.e., Lact-AM 400) at a nanotube concentration of 4.21 ± 0.13 μg/mL that are incubated with varying concentrations (i.e., 0, 0.40 ± 0.01, and 1.50 ± 0.20 μM) of proteins including (A) PNA, (B) Con A, (C) GNA, and (D) BSA for 5 min in PBS buffer solution.

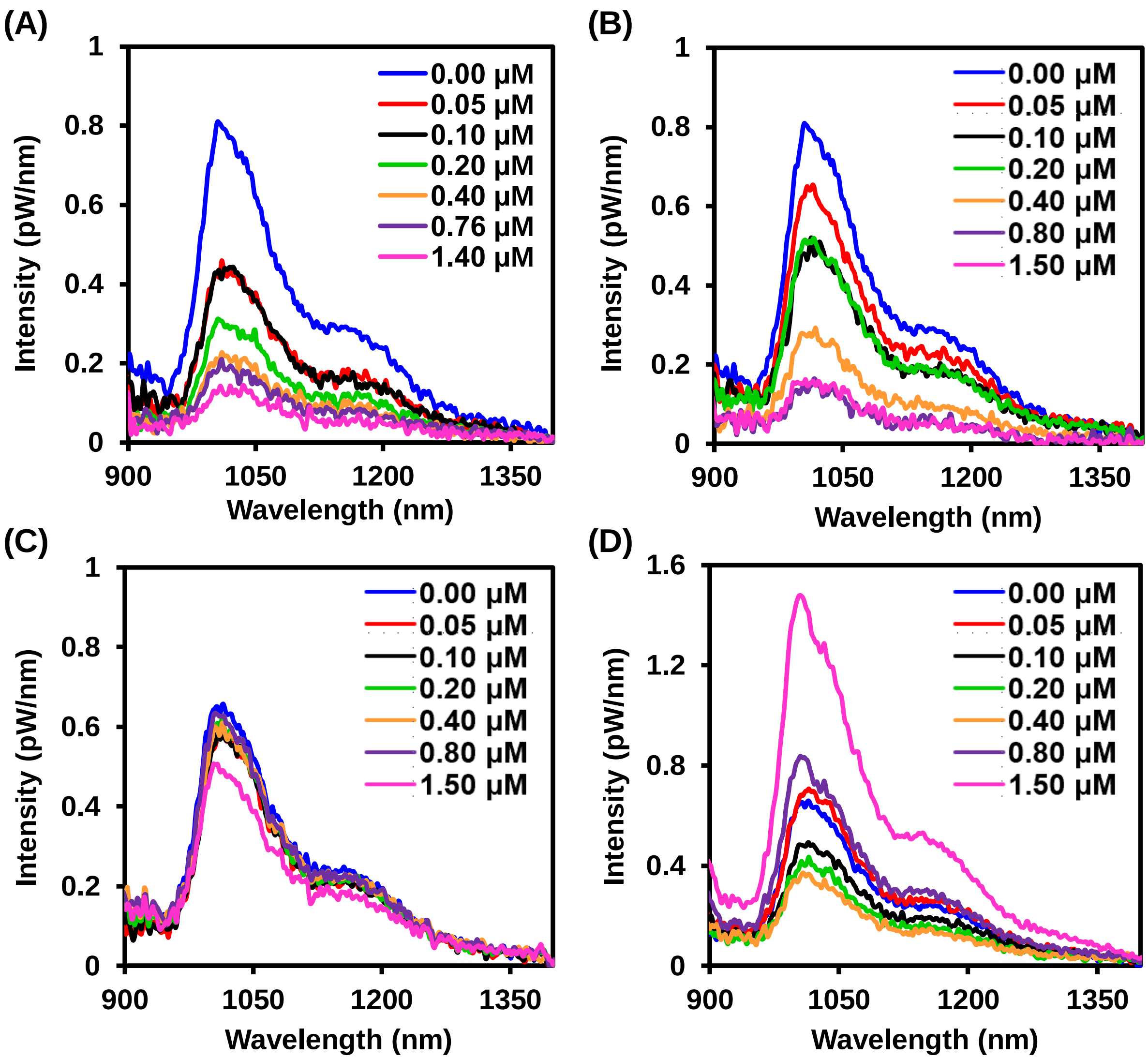

Figure S5. Representative NIR fluorescence spectra of Glyco-SWCNTs (i.e., Lact-AM 400) at a nanotube concentration of 4.21 ± 0.13 μg/mL that are incubated with varying concentrations (i.e., 0 − 1.50 ± 0.20 μM) of proteins including (A) PNA, (B) Con A, (C) GNA, and (D) BSA for 5 min in PBS buffer solution. A fixed excitation wavelength of 532 nm laser was used for NIR fluorescence spectra measurements.

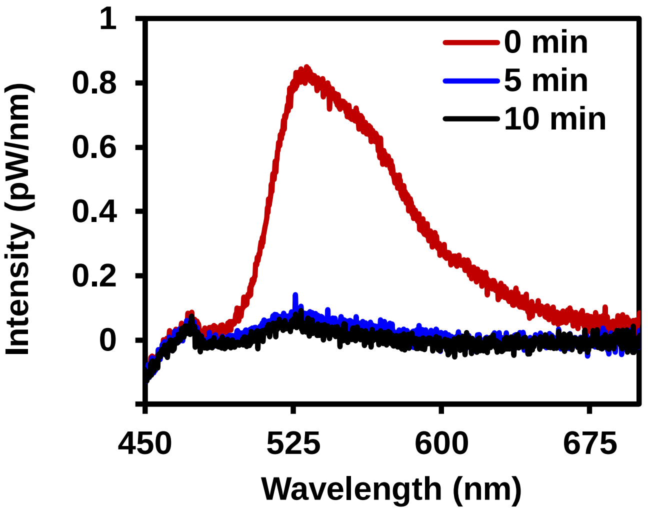

Figure S6. Visible fluorescence spectra of 38.64 ± 1.00 μg/mL (i.e., 0.32 ± 0.01 μM) PNA-FITC before (i.e., 0 min) and after (i.e., 5 and 10 min, respectively) being incubated with Glyco-SWCNTs (i.e., Lact-AM 400) at a nanotube concentration of 9.66 ± 0.74 μg/mL in PBS buffer solution. A fixed excitation wavelength of 408 nm laser was used for visible fluorescence spectra measurements.

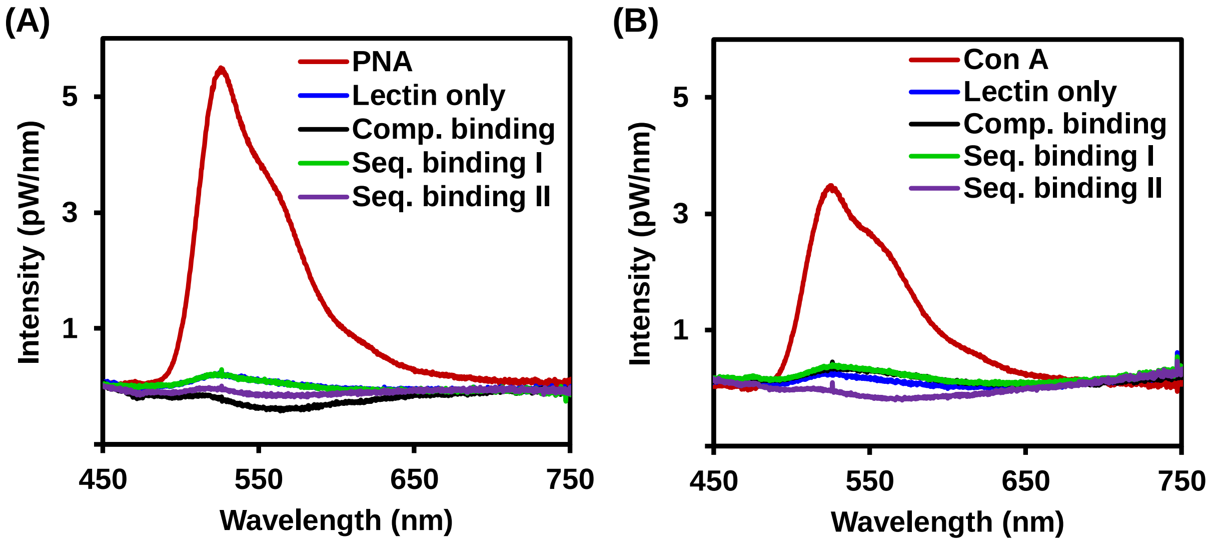

Figure S7. Visible fluorescence spectra of 0.77 ± 0.01 μM of (A) PNA-FITC and (B) Con A-FITC before and after incubating with Glyco-SWCNTs (i.e., Lact-AM 400) at a nanotube concentration of 4.21 ± 0.13 μg/mL in PBS buffer solution for 5 min, except that the Seq. binding I sample was incubated with nanotubes for a total of 10 min. Various incubation methods were employed including lectin binding, competitive binding (i.e., Comp. binding), and sequential binding I and II (i.e., Seq. binding I and II). A fixed excitation wavelength of 408 nm laser was used for visible fluorescence spectra measurements.

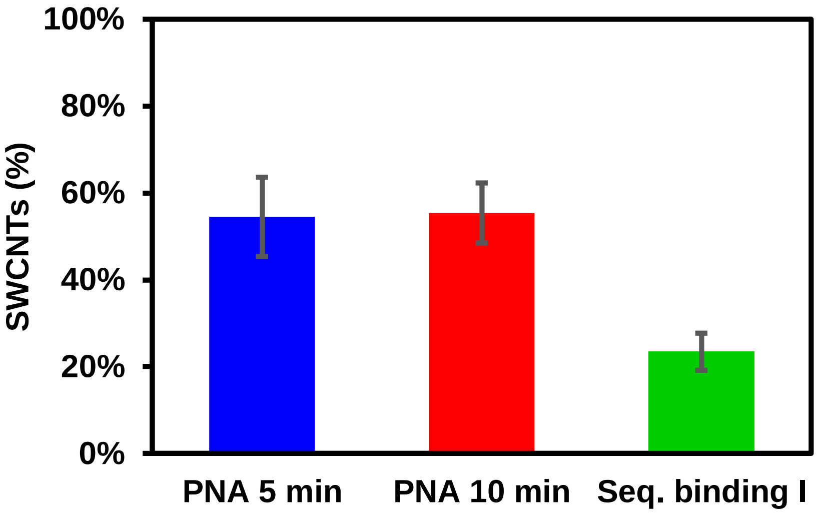

Figure S8. Comparison of the percentage of SWCNT remaining in the supernatant determined from the sample absorption at 780 nm after incubating Glyco-SWCNTs (i.e., Lact-AM 400) with PNA-FITC only in PBS buffer solution for 5 and 10 min as well as with PNA-FITC and free sugar β-lactose for a total of 10 min (i.e., Seq. binding I). The nanotube concentration is 4.21 ± 0.13 μg/mL and that of PNA-FITC is 0.77 ± 0.01 μM.

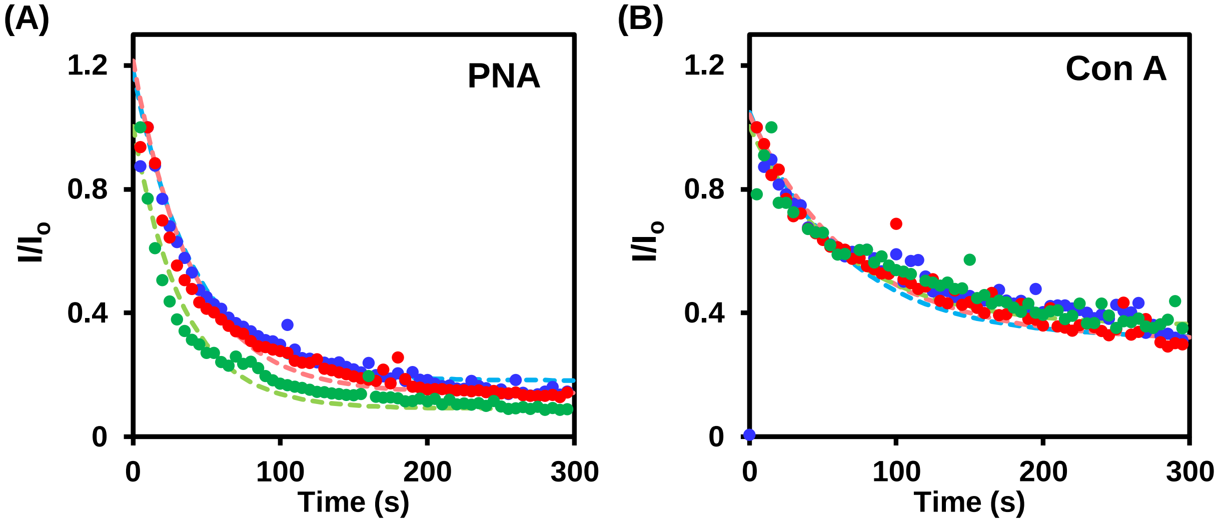

Figure S9. Measured (solid spheres) and exponential fits (dotted lines) of visible fluorescence kinetics of lectin FITC marker during interactions of (A) PNA and (B) Con A with Glyco-SWCNTs (i.e., Lact-AM 400) showing distinct responses in the emission intensity ratio change of FITC at 525 nm peak wavelength as a function of time. The concentration of lectins and SWCNTs are 2.23 ± 0.20 μM and 4.21 ± 0.13 μg/mL, respectively. A fixed excitation wavelength of 408 nm laser was used for visible fluorescence spectra measurements.

Table S2. Exponential fits of lectin FITC intensity ratio I/I_O at 525 nm vs. time for lectin interaction of Glyco-SWCNTs using $y = A\left(1 - e^{-x/t}\right) + B$.

Lectin	t (s)	A	B	R^2
PNA	38.42±3.26	-0.10±0.07	1.13±0.09	0.97±0.01
Con A	66.34±3.62	-0.70±0.04	1.03±0.02	0.92±0.03

*The emission intensity ratio of FITC marker at the 525 nm peak wavelength was denoted as I/I_o, where I_o and I are the magnitude of FITC 525 nm emission peaks before and after adding Glyco-SWCNTs (i.e., Lact-AM 400) at a nanotube concentration of 4.21 ± 0.13 µg/mL. The lectin concentration is 2.23 ± 0.20 µM. The time constant t obtained from the exponential fit corresponds to the $1/k$, where k is the rate constant. Standard deviations were obtained from repeats of three separate samples.

References

Schöppler, Friedrich, Christoph Mann, Tilman C. Hain, Felix M. Neubauer, Giulia Privitera, Francesco Bonaccorso, Daping Chu, Andrea C. Ferrari, and Tobias Hertel. 2011. "Molar Extinction Coefficient of Single-Wall Carbon Nanotubes." *J. Phys. Chem. C* 115 (30): 14682–86. https://doi.org/10.1021/jp205289h.
